# Rare Compound Heterozygous Frameshift Mutations in *ALMS1* Gene Identified Through Exome Sequencing in a Taiwanese Patient With Alström Syndrome

**DOI:** 10.3389/fgene.2018.00110

**Published:** 2018-04-18

**Authors:** Meng-Che Tsai, Hui-Wen Yu, Tsunglin Liu, Yen-Yin Chou, Yuan-Yow Chiou, Peng-Chieh Chen

**Affiliations:** ^1^Depatment of Pediatrics, National Cheng Kung University Hospital, College of Medicine, National Cheng Kung University, Tainan, Taiwan; ^2^Institute of Clinical Medicine, College of Medicine, National Cheng Kung University, Tainan, Taiwan; ^3^Center of Clinical Medicine, National Cheng Kung University Hospital, College of Medicine, National Cheng Kung University, Tainan, Taiwan; ^4^Department of Biotechnology and Bioindustry Sciences, National Cheng Kung University, Tainan, Taiwan

**Keywords:** Alström syndrome, *ALMS1* gene, ciliopathy, whole exome sequencing, childhood obesity, retinitis pigmentosa

## Abstract

Alström syndrome (AS) is a rare autosomal recessive disorder that shares clinical features with other ciliopathy-related diseases. Genetic mutation analysis is often required in making differential diagnosis but usually costly in time and effort using conventional Sanger sequencing. Herein we describe a Taiwanese patient presenting cone-rod dystrophy and early-onset obesity that progressed to diabetes mellitus with marked insulin resistance during adolescence. Whole exome sequencing of the patient's genomic DNA identified a novel frameshift mutation in exons 15 (c.10290_10291delTA, p.Lys3431Serfs^*^10) and a rare mutation in 16 (c.10823_10824delAG, p.Arg3609Alafs^*^6) of *ALMS1* gene. The compound heterozygous mutations were predicted to render truncated proteins. This report highlighted the clinical utility of exome sequencing and extended the knowledge of mutation spectrum in AS patients.

## Introduction

Alström syndrome (AS; OMIM 203800) is a rare autosomal recessive disorder characterized by early-onset blindness due to cone-rod dystrophy, juvenile obesity followed by marked insulin resistance and type 2 diabetes mellitus, and progressive sensorineural hearing impairment that usually takes place within the first year of life (Marshall et al., 2007a). Moreover, a certain portion of AS patients also presents liver, kidney, neurological, cardiac, and pulmonary diseases (Marshall et al., 2005). No disease specific treatment is as yet available, whereas early morbidity and mortality are expected in affected patients.

The causative gene of AS has been recently ascribed to *ALMS1*, which harbors 23 exons and encodes a 461-kDa protein (ALMS1, centrosome and basal body associated protein) involved in ciliary functions (Hearn et al., 2002). Although the multi-organ pathogenesis of AS has not been fully delineated, it is suggested that truncated or dysfunctional proteins disrupt cell cycle regulation and intracellular trafficking (Girard and Petrovsky, 2011). Clinical diagnosis is based on observation of cardinal features. However, varied age of onset and evolutional severity of manifestations may be due to the allelic and expressive heterogeneity of AS and thus defer the diagnosis particularly in patients with only part of clinical features that are also shared by other ciliopathies, such as Bardet-Biedl syndrome (Marshall et al., 2007a). Under such a circumstance, confirmatory mutation analysis of *ALMS1* is required and usually laborious by conventional Sanger sequencing, given the size and mutation spectrum of *ALMS1* gene along with other disease associated genes involved in the ciliopathies (Marshall et al., 2007b). This hardship can be resolved by the application of next generation sequencing techniques, which are now available for the identification of genetic variants comprehensively (Bamshad et al., 2011). Here, we reported a Taiwanese patient with AS features and identified a novel mutations in *ALMS1* through whole exome sequencing (WES).

## Clinical report

A 16-year-old Taiwanese boy, born uneventfully to Taiwanese parents, measured 147 cm in height and 55 kg in weight with a body mass index 25.2 kg/m^2^. He presented hyperphagia and rapid weight gain at the age of 5 months when he measured 10 kg (>97th %) and 69 cm (>75th %). Meanwhile, he received diagnosis of bilateral nystagmus that progressed to nearly blindness at the age of 4. Funduscopic examinations revealed retinitis pigmentosa. He was hospitalized at the age of 11 because of diabetic ketoacidosis and acute pancreatitis. Examinations also revealed hypertriglyceridemia, severe fatty liver, and renal insufficiency. The initial metabolic survey found that marked insulin resistance and pancreatic insufficiency with the homeostatic model assessment for insulin resistance (HOMA-IR) index was 83.5 [a value greater than 2.6 may indicate insulin resistance in adolescents (Burrows et al., 2015)] and HOMA-ß 78.2% [a value greater than 100% may indicate reserved insulin secretion (Matthews et al., 1988)]. He was started on insulin therapy since then and the daily requirement progressively exceeded 260 IU/day with the presence of remarkable acanthosis nigricans within 5 years' time. Updated endocrine investigation showed an optimal increase of C-peptide secretion in response to intravenous glucagon stimulation. Echocardiography revealed a borderline left ventricular dilatation. The intellectual and hearing ability were both unaffected.

## Methods

Informed written consent was acquired from the patient and his parents, as the entire procedure of this study was approved by the Institutional Review Board of the National Cheng Kung University Hospital (B-BR-104-063). The proband's genomic DNA was extracted from peripheral blood collected in EDTA-containing tubes. SureSelect QXT All human exon V6 (Agilent), which targeted 60 Mb of the exonic regions, was applied to construct the exome library that was then sequenced on Illumina NextSeq500 platform. We aligned the sequence reads to human genome reference Hg19 using Novoalign (www.novocraft.com) and identified single nucleotide variants and small insertions and deletions using Genome Analysis Toolkit 3.4 (GATK; www.broadinstitute.org/gatk). The sequence variants were annotated with SeattleSeqAnnotation (snp.gs.washington.edu/SeattleSeqAnnotation138) and novel variants were filtered against 1000 Genomes, dbSNP, and Genome Aggregation Database (gnomad.broadinstitute.org). Sanger sequencing was finally used to confirm the mutation.

## Results

**Figure 1:**
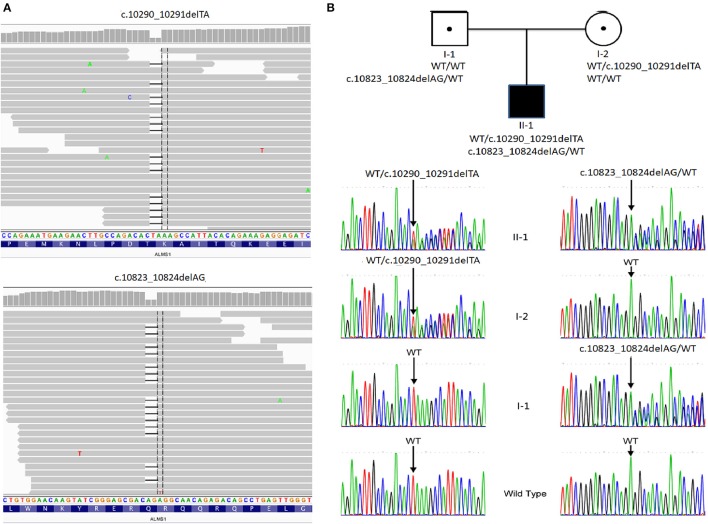
Pedigree and mutation analysis of the family. **(A)** Alignment of exome sequences to Hg19 showing 2-base pair deletion in exon 15 and exon 16 of *ALMS1*. **(B)** The proband (II-1) carried compound heterozygous mutations: c.10290_10291delTA inherited from his mother (I-2) and c.10831_10832delAG from his father (I-1). WT, wild type.

**Figure 2:**
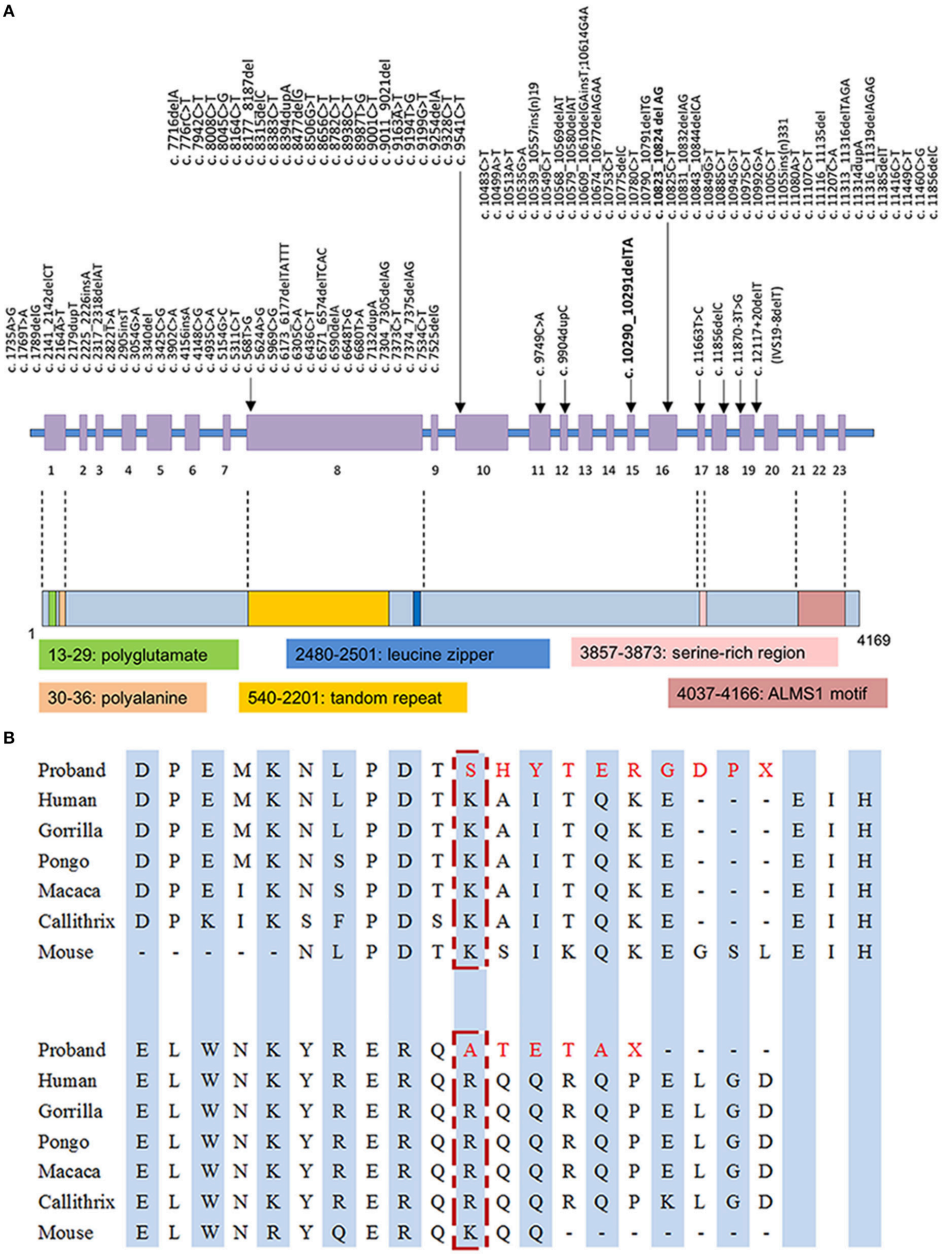
**(A)** Mutation spectrum of *ALMS1* and domains structure of ALMS1. The frameshift mutations identified in the proband in this study are in bold. **(B)** Conserved amino acid sequences of ALMS1 (amino acid 3431 and 3609) and the predicted truncated ALMS1 caused by the frameshift mutation identified in this proband.

With the average coverage of 40.1X on targeted regions, we identified 28,967 novel genetic variants in the exome sequencing. The summary of variants identified was listed in Table 1. Two pathological mutations in *ALMS1* were identified: chr2:73786171delTA (c.10290_10291delTA) in exon 15 (Figure 1A, upper panel) and chr2:73799829delAG (c.10823_10824delAG) in exon 16 (Figure 1A, lower panel). These mutations were further confirmed by Sanger sequencing (Figure 1B) and the results showed that c.10290_10291delTA (p.Lys3431Serfs^*^10) was maternally inherited and c.10823_10824delAG (p.Arg3609Alafs^*^6) was paternally inherited. These mutations resided in a conserved stretch of amino acids and led to truncated proteins lacking the ALMS motif (Figures 2A,B).

**Table 1:** Summary of whole exome sequencing on the patient.

Total captured region size	1,749,161,265
% of captured regions with coverage >15	86.2
Mean coverage of target region	40.1
Total number of SNPs	310,919
Total number of INDELs	29,200
Total number of novel GVs not listed in dbSNP	28,967
Total number of SNPs in a panel of ciliopathy-related genes	12
Total number of INDELs in a panel of ciliopathy-related genes	3

*SNP represents single nucleotide polymorphism; INDEL, insertions and deletions, GV, genetic variants*.

## Discussion

In this report, we described a Taiwanese patient presenting clinical features compatible with AS, except the absence of hearing impairment. Using WES, we identified compound heterozygous mutations in the exons 15 and 16. Both affected alleles were frameshift mutations that were predicted to cause premature stop codon downstream and render truncated ALMS1 protein.

The ALMS1 protein consists of 4,169 amino acids and contains several domains including a potential signal peptide at residues 211–223, a leucine zipper at residues 2480–2501, and an ALMS motif at residues 4035–4167 (Collin et al., 2002; Hearn et al., 2002). The exact molecular role of these motifs is not completely elucidated, although truncated ALMS1 proteins have exhibited perturbed effects on intracellular localization, microtubular organization, cell cycle regulation (Hearn et al., 2002, 2005; Girard and Petrovsky, 2011). In our patient, truncated proteins, if transcribed from the observed mutations p.Lys3431Serfs^*^10 and p.Arg3609Alafs^*^6, are presumed to lack the ALMS motif that is essential in co-localization with centrioles and basal bodies (Knorz et al., 2010). Given that *ALMS1* is ubiquitous expressed in human tissues, low abundance or functionality of transcripts may contribute to the multi-organ pathogenesis in AS, such as metabolic and neurosensory disorders (Collin et al., 2002; Hearn et al., 2002; Girard and Petrovsky, 2011). However, phenotypic variability exists in the same *ALMS1* mutated spots, even within the same family (Titomanlio et al., 2004). Our patient did not present typically sensorineural hearing impairment, which can be explained by the prior assumption that genotype-phenotype correlation of *ALMS1* mutations may be modified by other modifier genes or environmental factors (Collin et al., 2002; Marshall et al., 2007b).

A wide array of nonsense and frameshift mutations has been identified in coding regions of *ALMS1* gene with potential hotspots preferentially located in exons 8, 10, and 16 (Figure 2A) (Marshall et al., 2007b; Ozantürk et al., 2015). Ethnicity remains as a strong contributor to the distribution of genetic variants. However, the skewed clustering of mutations may be affected by genetic founder effect and consanguinity. In East Asian descents, most reported mutations were disperse within the aforementioned cluster of exons but distinctive in location and structure (Liang et al., 2013; Marshall et al., 2015). Only a few variants have been reported more than once in East Asian populations, such as c.11116_111134del recurring in Taiwanese families (Marshall et al., 2007b; Lee et al., 2009). The rare variant c.10823_10824delAG has been exclusively reported in East Asian descents with an estimated allele frequency of 2.53 × 10^−5^ in the up-to-date Genome Aggregation Database^1^. Therefore, we assumed a genetic founder effect on this rare pathological variant. Obtaining genetic diagnosis of clinical patients in under-genotyped populations can expand our knowledge about this disease entity and relevant functionality of *ALMS1* gene. From practical perspective, screening for these mutations in suspicious cases will be a feasible diagnostic step if there are sufficient data regarding the prevalence of *ALMS1* mutations in the local population. Otherwise whole gene sequencing is usually required to fully capture the genotypes.

Technological advancement in high throughput sequencing has met clinical needs and thus hastened the identification of novel pathological variants in *ALMS1* gene (Bamshad et al., 2011; Ozantürk et al., 2015). As compared to conventional Sanger sequencing, WES provides an effective and efficient alternative method that aids genetic diagnosis especially in cases with overlapping features among a certain number of differential diagnoses enlisted within the spectrum of ciliopathy. Moreover, the clinical utility would be significantly promoted within the near future as the cost of WES is expected to drop below that of Sanger sequencing, when reading a sizable coding region of target gene is required. Our report highlights the value of WES in providing genetic diagnosis in rare diseases.

## Author contributions

M-CT and P-CC conceived the study; M-CT, Ye-YC, and Yu-YC collected clinical information; H-WY, TL, and P-CC conducted the bioinformatics analysis; M-CT drafted the manuscript and P-CC supervised the entire study. All the authors approved the final version of manuscript.

### Conflict of interest statement

The authors declare that the research was conducted in the absence of any commercial or financial relationships that could be construed as a potential conflict of interest.

## Footnotes

^1^The Genome Aggregation Database. Available online at: http://gnomad.broadinstitute.org/about (Accessed January 18, 2018).
